# Qualitative document analysis on Iranian contents and trends of population policies: Lessons learned and avenues for future

**DOI:** 10.1016/j.heliyon.2023.e17377

**Published:** 2023-06-21

**Authors:** Reyhane Izadi, Mohammad Amin Bahrami, Yaser Sarikhani, Peivand Bastani

**Affiliations:** a Department of Health Care Management, School of Management and Information Sciences, Shiraz University of Medical Sciences, Shiraz, Iran; b Health Human Resources Research Center, Department of Health Service Management and Health Economics, School of Health Management and Information Sciences, India; c Research Center for Social Determinants of Health, Jahrom University of Medical Sciences, Jahrom, Iran; d College of Health and Human Sciences, Charles Darwin University, Alice Springs, NT 0870, Australia; e Faculty of Health and Behavioral Sciences, School of Dentistry, University of Queensland, Brisbane, QLD, 4072, Australia

**Keywords:** Qualitative document analysis, Population policies, Political requirements, Iran, policy analysis

## Abstract

Population-related policies are among macro-strategic policies considered by all governments in the world. To achieve the desired population structure, it is first necessary to identify the general policy approach over time. This article aims to identify the main requirements of population policies during the last 70 years in Iran. This is a qualitative content analysis study conducted via the analysis of all relevant national policy documents from 1951 to 2022. To retrieve the relevant documents, we searched the official website of eight policymaking bodies in Iran. After identifying the documents, their eligibility was evaluated using Scott’s method, and as a result, 40 documents were selected for analysis. Finally, we used a qualitative content analysis to synthesize the data using MAXQDA version 10. The findings showed that the political requirements for population reduction can be classified into four main themes of “Religious, scientific, and legal infrastructure”, “Changes in the rules”, “Institution building, programming and division of tasks”, and “Information and service provision”, with 11 sub-themes. Furthermore, the political requirements for an increasing population can be divided into six main themes of “Education & acculturation”, “Legal dos, and don’ts”, “Financial and non-financial support for families”, “Structural and information infrastructure”, “Health services”, and “Stewardship”, with 30 sub-themes. In this study, by an all-round look, and the analysis of policies of the last 70 years of Iran, it was determined how the population policies originate from the political-cultural background of society, and provide the ground for the changes in the cultural-social, political, and economic structures and as a result demographic change. In other words, the main requirements to formulate the population increase and decrease policies in Iran as a country with golden experiences to implement the population policies were shown; which can be helpful as a guide to formulate the population policies in Iran and provide a model for successful policy making in the countries with a similar background to Iran.

## 1 Introduction

In today’s world, all modern governments are concerned with population, and managing people’s social and individual life lives. For this reason, they follow different policies to address demographic issues [1]. The nature of population policies in health, especially fertility, and childbearing is complex. Therefore, the decision of families, and policymakers regarding procreation and family size has been controversial and varied over time [2]. Fertility is one of the most important natural phenomena, and one of the determinants of the population growth [3], for which some countries apply incentive policies and others penal policies [4].

Population growth has always been a contentious issue with serious proponents, and opponents in terms of its economic, social and environmental impacts [5]. These disagreements were reflected in anti-natalist and pro-natalist policies [6]. The demographic change is seen as an important, and influential variable in all development planning [2]. Furthermore, population growth is considered a key component of sustainable development [7]. In this context, the population policies are established to control the reproduction, and sexual life of people [1]. Moreover, recognizing population dynamics, and related factors is one of the essential imperatives of development programs in today’s world. This is particularly important for developing countries, which are most concerned about development and growth [7]. In these countries, the demographic change is doubling the challenges to achieve social, and economic development [8].

The evolution of reproductive health in Iran shows that the family planning program was suspended shortly after the Islamic revolution and childrearing was encouraged. This policy shift reflected the socio-political atmosphere created by the revolution, and the Iran-Iraq eight-year war (1980–1988). Finally, four months after the revolution, the Ministry of Health received fatwas from the founder of Islamic Revolution that it is permissible for couples to use modern contraceptives [9]. On the other hand, International Conference on Population and Development (ICPD) in 1994 led to creating a new paradigm that placed population issues, and comprehensive reproductive rights within, rather than alongside, sustainable economic, and environmental development plans [10]. As a result of this conference, young women in developing countries have become increasingly in need of programs related to sexual and reproductive health. And finally, as a result of these political and social developments in Iran and the world, family planning in Iran was put on the agenda. This family planning program was implemented with specific plans to achieve the three goals of preventing very early pregnancies, spacing among the pregnancies by three-year intervals, and encouraging women not to have more than three children. This program increased the prevalence of contraception, and contraceptive use increased from 49.9% in 1989 to 73.8% in 2000 [11]. Iran is one of the countries that seriously implemented population policies, especially birth control, in the late 20th century [1] and reduced the fertility rate from 6.5 per woman in 1986 to 2.5 in 2010 [12]. Besides, the evidence indicates that the maternal mortality rate in Iran has declined from 40 deaths per 100,000 live births in 1990 to 14 in 2015, indicating a 1.36% decrease on average; It means progress beyond the fifth goal of the United Nations Millennium Development Goal [11,13]. Although Iran has achieved significant achievements in reproductive health, and its family planning program was introduced as one of the most successful programs in the world, it still faces serious challenges that require systematic efforts. Improving the role of non-governmental organizations, and improving community-based programs to support reproductive health, developing teenage-friendly and male-friendly services, are among the issues that need more attention [11]. There is a limited access to legal abortion currently in Iran, which is one of the biggest obstacles for reproductive health. It is difficult to obtain accurate official data on abortion rates in Iran due to its difficulty and the fact that it is often performed illegally. Studies in Iran indicate about eight abortions annually per 1000 women of reproductive age [11,14].

Along with the obstacles related to reproductive health, economic pressures in recent years have led to a decrease in fertility in Iran. After overcoming economic problems, fertility can be expected to increase again. However, other deterrents, such as urbanization, improved education, especially for women, and a higher marriage age will certainly lead to greater reductions in fertility in the future. Moreover, when a family has better financial security, it is expected that it will care more about the quality of life of its children than the size of their family [12]. As a result, in terms of the extreme complexity of factors affecting the population issue, governments should formulate population policies to manage the population growth, and structure by using a multi-sectoral approach [15].

Population policies in recent years in Iran indicate that the government has tried to curb the population decline by abolishing the family planning program, and offering married couples various social incentives [16]. A statement on general population policy at the highest national level was issued in May 2014 to develop a comprehensive population policy. The statement includes several goals, such as achieving a fertility rate equal to or above the replacement level, facilitating and promoting family formation and childbearing, strengthening the foundations and stability of the family, and promoting, and establishment of the Islamic-Iranian lifestyle [17]. Despite the importance of new population policy in Iran, the evidence indicates that there is fragmentation, and inconsistency between actors with different powers and positions in this field; and it seems that in new period of population policy reform, these actors have not mobilized together as expected, as in the past [18]. On the other hand, although the evidence indicates that Iran has a brilliant history in the implementation of population policies [11,19,20]; and studies were conducted on this issue [1,16], there is still no comprehensive qualitative research that has analyzed all of Iran’s population policies (both population increase and decrease) over the long years (before and after the revolution). Considering the successful experiences of Iran in population policies [11], it seems that it is necessary to determine the policymakers' approach to the population problem over time to achieve a desirable population system, and formulate a comprehensive package of population policies that are compatible with all elements of society [[21], [22], [23]].

This research has tried to be helpful to formulate new population policies of Iran by comprehensively showing the issues of concern to policymakers over the years and be a guiding light for taking action to build the future. Furthermore, by presenting Iran’s population policies as a successful model [11], this study provided a deeper understanding of effective policy-making for countries with similar contexts. This research clarifies how in a developing country, by adopting different types of population policies, it is possible to provide the ground for changes in social, cultural, economic, and political structures, and as a result, fundamental changes in the population structure. To provide a comprehensive picture of the political approaches to population management in Iran in the last 70 years, this article analyzed all national documents related to population management. This research clearly answers that, firstly, “have Iran’s upstream documents in different periods of the last 70 years unilaterally recommended population increase or population decrease?”; and secondly, in Iran, as a successful country in the implementation of population policies, “what have been the political strategies/requirements to increase, and decrease the population?”. By determining political requirements for population increase and decrease, which is the most important goal of this research, it is explained how experience can guide the future. This study takes us on a journey from the past to the edge of tomorrow, the journey that starts with the first politico-demographic decisions in Iranian history, and ends with our responsibility to future generations.

Using qualitative documents analysis, we answered these research questions. In this method of qualitative analysis, documents are reviewed and evaluated in a systematic manner. Various forms of documents can be analyzed using this approach. Moreover, because the documents are not affected by the study process, this method solves the problem of reflexivity which is a great concern in other qualitative studies. Qualitative documents analysis ensures the stability of data, and it covers a long period of time, many events, and many situations [24].

## 2 Literature review

In general, the evidence indicates that the economic climate is a factor affecting fertility in different ways. In Europe, the fertility rate has decreased during the economic recession [25]. Moreover, in Asian countries, economic growth had a positive effect on fertility rate [26]. Studies have shown that policies that reduce childcare burdens on mothers have a greater impact on childbearing decisions [27]. It was found that in Japan, the main reason for fertility reduction after 1970 is in terms of the decline in the marriage rate, which is mainly in terms of the adoption of policies that have led to increased employment opportunities for women [28]. Population policies in the Middle East should take into account the five main factors contributing to the declining total fertility rate, according to a systematic review. These factors include health care (increased beds/hospitals, increased men’s participation in sexual health practices, etc.), social factors (increased urbanization, reduced rate of early marriage, etc.), cultural factors (Changes in women’s attitudes towards employment, weakening of traditional values of societies concerning child-raising, etc.), economic factors, high costs of marriage and child-raising, inflation, etc.), and political factors (the establishment of restrictions for polygamy, governments' direct support policies for family planning, etc.) [29]. A study was conducted by the purpose of determining the main solutions to increase fertility from the perspective of policy makers in Iran, and it showed that the main theme is “improving infrastructures”, which includes 6 categories of removing obstacle to marriage, improving women’s working practices, care services and training for the future generation, training and consulting, research, improving services [30]. In order to support Iran’s new population increase policy, family planning services are restricted, maternity leave is increased, and natural childbirth is encouraged [2,31]. Although many evidences indicate Iran’s brilliant experience to implement the population policies [1,11,16], there are currently challenges, such as lack of experts in reproductive health policymaking, lack of sufficient evidence to make decisions, lack of attention to spatial planning infertility policy making, and lack of ideological-based reproductive health policymaking in the process of population policymaking [2].

## 3 Data and methods

### 3.1 Description of the context

Located in the Middle East, the Islamic Republic of Iran covers an area of 1,648,195 km^2^, making it the fourth largest country in Asia and the second largest in Western Asia. Iran is a culturally diverse country, made up of numerous ethnic and linguistic groups united by a common Iranian nationality [32]. The country’s population has grown rapidly since 1956, from around 19 million to over 84 million by 2020, making it the 17th most populous in the world. However, over the past two decades, Iran’s fertility rate has dropped dramatically from 6.5 per woman to just a little more than 2, resulting in a population growth rate of around 1.39% in 2018. Based on the statistics published in 2022, the country’s population was 85,891,580, the sex ratio was 1.03 (43,558,927 males and 42,333,787 females), the life expectancy of the general population was 70.1 years (68.6 for males and 71.6 for females), and the population density was 52.1 per km^2^ [33]. Iran has experienced a political revolution in 1978 and a local war in 1980, both of which can significantly affect the country’s population growth trends.

### 3.2 Study design

This is a qualitative content analysis study that applies a narrative approach to the analysis of national policy documents [24,34]. Qualitative research can be conducted using previous available data such as documents and other textual data, according to Ref. [35]. The textual data and documents in this type of research refer to government instructions and circulars, such as official documents, policies and programs issued and periodic reports [35]. At the same time, Krippendrof (2018) believes that documents or other textual data, such as government directives and guidelines, official documents, policies and programs, and periodic reports can be analyzed through a hermeneutical approach in a five-step process, including access to data and documents, checking the validity of documents, comprehension of the documents, data analysis, and summarizing the information in the form of extracted themes [36].

Regarding the national documents, legislation, and laws can be considered as the basic guidelines for policymaking, and because their content can reveal the main views and strategies of the country in a given field, to answer the current research question, qualitative document analysis was used to identify the main political requirements in Iran’s population policies.

### 3.3 Data collection

To retrieve the most relevant documents, we systematically searched the official websites of eight national policymaking bodies, including the Islamic Parliament Research Center [37], Ministry of Health and Medical Education [38], Plan and Budget Organization [39], Statistical Center of Iran [40], Ministry of the Interior [41], Laws and Regulations Portal of Iran [42], National Organization for Civil Registration [43], Hawzah Information Database [44].

To collect the data, we searched mentioned websites using related phrases and keywords, including “fertility”, “infertility”, “population growth”, “family planning”, “birth control”, “marriage age”, “family excellence”, “childbearing”, “aging”, “youth of the population”, and “Iranian-Islamic lifestyle”. We have considered the time period from 1951 to 2022 for the search to provide a comprehensive analysis of national population policies before and after the Islamic revolution in Iran (1978). The set of documents chosen was selected because all Iranian political considerations regarding the population issue can be found in the organizations mentioned and related documents, laws and legislation, which can provide us with a comprehensive source of information to answer our research question. For the initial review of available documents, and to create a logical trend and avoid the loss of relevant information, the research team has prepared a data collection form. Based on the study objectives, the document name, the location of document retrieval and brief descriptions of the document content were included in this form.

### 3.4 Validation method

To ensure the eligibility of data, we used Scott’s 4-step method to determine authenticity, credibility, representativeness, and meaningfulness [45]. To verify authenticity, we first examined the source of the documents, so that at this stage only the documents that were approved by parliamentarians and government officials were considered authentic. In the second step, to check credibility, only documents that are not misleading and show no organizational or personal conflict of interest were admitted. In the third step, representation meant that the retrieved documents indicated general policies or keywords that were determined based on the purpose of the study. Finally, in the last step, meaningfulness meant that the document was comprehensive, and the content and face validity of the document were approved. Applying Scott’s four steps, documents that fully covered all four criteria were selected for final analysis [46]. In this regard, a total of 40 documents out of 56 documents were included in seven main categories (see Table 1 and supplemental Table S1) and other documents were excluded for lack of at least one of Scott’s quadruple criteria.

**Table 1:** Final categories of included documents.

No	Final Categories of Included Documents	Number of Documents
1	Social economic development plans (After and before the Islamic Revolution)	9
2	National macro-plans for population management	9
3	Laws and regulations affecting the population management	11
4	Serious political recommendations of the leaders of the Islamic Republic of Iran	2
5	National land management document	1
6	Duties of communication of executive bodies by the cabinet	1
7	International contracts, agreements, and programs in population management	7

### 3.5 Data analysis

We used a qualitative content analysis to synthesize the data. Content analysis deals with inductive and deductive approaches to data analysis. To find the initial concepts deductively, one of the researchers (YS) screened all the eligible retrieved documents based on the available concepts in the literature. In order to achieve this goal, the research question, keywords, and concepts from previous studies were used. The initial outcome of this deductive approach was exploring two concepts for the dominant categorization of population policies as follows: policy requirements in family planning and birth control (population decrease), and family excellence and childbearing (population increase). Then, in terms of the inductive approach, another researcher (RI) used a thematic method for moving from data (content of the documents) to initial and final codes and more intellectual concepts like subthemes and themes. This thematic six-step is elaborated as follows [47]:

Stage 1) Familiarization: In this stage, the authors first read all texts several times to familiarize themselves with the content, then reviewed them again to determine the meaningful units of the text using an implicit approach. At the same time, we tried to agree on the meaning of the phrases “population increase” and “population decrease” in the population policy documents. Thus, we searched the content of the documents for similar topics and concepts such as fertility, infertility, population growth, sterilization, and abortion.

Stage 2) Generating codes: In the second stage, using the inductive approach and the open coding method, we started creating codes in such a way that, after finding relevant phrases or words, we assigned a suitable code to each determined phrase. The initial codes have been revised many times to create new codes and merge repeating codes. Accordingly, the final codes were generated. In this stage, we used MAXQDA version 10.

Stage 3) Generating themes: In the third stage, we again checked the final codes again to identify the pattern among them, then we tried to merge the related final codes together to develop the sub-themes and then we reached the main themes. In this step, the relationship between the main themes, and sub-themes is clarified.

Stage 4) Reviewing themes: In the fourth stage, we had to make sure that the resulting themes were an accurate representation of the data. In fact, we compared our themes with reference to the data set. We revised and modified the generated themes on two levels. The first level included the extracted codes and the second level included the entire dataset. In other words, we read all the summaries collected on each theme and modified it as needed. We continued this process until we were sure that a consistent pattern was established among summaries. We then went via the same process at the level of the entire dataset, until we were sure that the themes explained the dataset correctly. By repeating this process, we finally created a satisfying thematic map that matched the dataset.

Stage 5) Defining and naming themes: In this stage, the research team discussed the names of the themes based on their internal nature and the aspects of the data contained in the themes. Therefore, an attempt was made to give the themes understandable and short names.

Stage 6) Producing the report**:** In this regard, we have provided a detailed description of the analysis method. According to ethical considerations, the names of individuals have been removed from the content of the policy. The research team had no conflict of interest in performing the qualitative analysis.

Finally, the codes generated by inductive and deductive analysis then were discussed and confirmed by all research team members.

### 3.6 Rigor and trustworthiness

The criteria developed by Guba and Lincoln, including credibility, dependability, transferability, and confirmability were used to ensure the rigor and trustworthiness of the study [48]. To ensure the credibility, the research team had a prolonged engagement with the documents and used a peer check method. To improve the dependability, we tried to clearly document the research process. Besides, the entire study process was also reviewed by two reviewers. To ensure the transferability, a thick description of the method is provided. In order to improve confirmability, two external experts were asked to confirm the precision of the study process. A number of memos were written throughout the study in order to ensure reflexivity and to minimize the effects of presuppositions.

## 4 Results

**Fig. 1:**
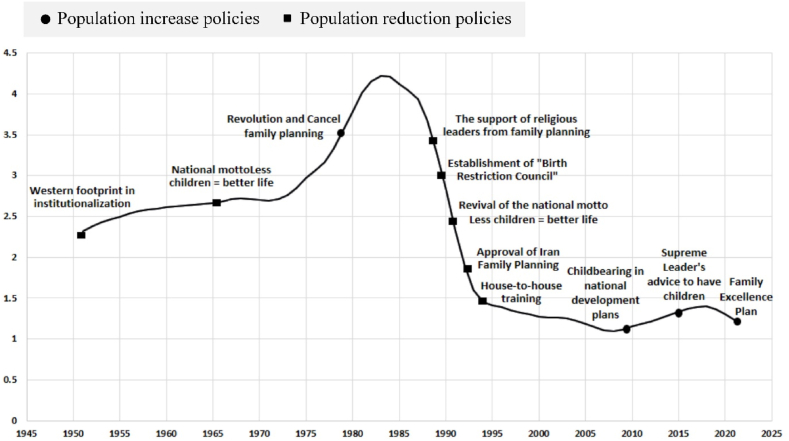
Iran’s population growth rate and population policies in the last 70 years.

In general, it was found that when policy makers focused on population increase, they considered six main themes and 30 sub-themes, and when they focused on population decrease, they considered four main themes and 11 sub-themes as population policy requirements. In addition, the results showed that socioeconomic, political, and cultural factors strongly influence the population policies and finally the demographic structure of Iran. The pattern of Iranian population policy over the past 70 years showed that the country has had four main periods of population management and during each period the general approach to population policy has changed (Fig. 1). For a better understanding of main themes, and sub-themes extracted in this research, each population policy period and the reasons for the policy shift in that period are briefly mentioned below.

• The first period of population reduction (1952–1978), before the Islamic Revolution, the first national population management policy: State intervention in the Iranian family domain started in the first quarter of the 20 lh century when the Pahlavi dynasty came to power. New civil law codes were enacted in 1928 as part of the new government’s modernization process, which reorganized the judiciary administration traditionally under Islamic religious leadership. While historically most family affair matters were handled within the framework of religious tradition and local practice, new law relegated all of these matters to a secular judicial system. Although state intervention in family affairs continued to expand into the first half of the 20th century, intervention in family size was not taken seriously until the 1960s [49]. Finally, in response to the challenges of rapid population growth, and the limitations of economic growth, and following the improvements made in the legal status of women in issues, such as polygamy, the right to divorce, custody of children, and employment opportunities, after some flirtation with a pronatalist policy, the Imperial Government of Iran adopted a national family planning program in 1967 [19,49].
• The first period of population increase (1978–1988), the first decade of Islamic Revolution, the repeal of all population reduction policies and scattered implementation of population increase plans: By the victory of the Islamic Revolution in 1978, an explicitly theocratic system based on the Shiite notion of Wilayat al-Faqih (rule of the jurist) was established, in which Shia ulama (religious leaders) were directly involved in policy-making and day-to-day administration of the country [9]. Iran’s new leaders viewed the family planning program as an imperialist tool to maintain the dominance of West over the “Third World,” and in particular, its Muslim population [19]. According to religious leaders, early marriage and family formation were promoted as fundamental Islamic values following the victory of the Islamic Revolution and the family planning program was suspended. This policy shift was a reflection of the socio-political atmosphere created by the Islamic Revolution, which was reinforced by eight-year Iran-Iraq war (1980–1988) [9].
• The second period of population reduction (1988–2010),from the second decade after the Islamic Revolution, the beginning of the most severe population control plans: As a result of the decisions of an explicitly theocratic system, about four months after the end of Iran-Iraqwar, the Ministry of Health of Iran received fatwas from the founder of the Islamic Republic and other Grand Ayatollahs that using the modern contraceptives by couples is not against Islamic tenets [9]. On the other hand, after International Conference on Population and Development (ICPD) in 1994, a new paradigm was created in the world and reproductive rights were considered an essential part of development programs. Consequently, the demand for reproductive and sexual health programs in developing countries increased, and Iran was no exception [10]. The Iranian government approved a family planning program in 1993 to reduce fertility in response to these socio-political changes in Iran and the world. However, there are evidences that the declining fertility in Iran started before the implementation of the family planning plan. It appears that this collective decision of the population to reduce the birth rate was affected more by socio-economic conditions than by the accessibility of family planning services. Economic problems, unemployment, increasing child rearing costs, women’s autonomy, and access to education were all factors affecting fertility [30].
• The second period of population increase (2010–2022), since the tenth government after the Islamic Revolution, population increase policies with an emphasis on childbearing: In recent years, population aging and low total fertility rate and their adverse effects have given rise to various economic and political considerations in the countries of Middle East and North Africa region and have led to changes in the population policies of some countries, including Iran [29]. Iran changed its policy when it anticipated a shift in age structure and possible population decline, as well as to prevent the spread of the “western lifestyle” based on small families. Based on the statements of the leader of the Islamic Republic, due to the importance of the issue of population to achieve national authority, and considering the country’s young population as an advantage to achieve the socio-economic development, and to compensate for the decrease in population in recent years, new population policies should be implemented with the overall goal of increasing Iran’s population from nearly 80 million people today to 150 million people by 2050 [50]. These statements, like a radical voltage, forced Iranian managers and policymakers to revise the former population policy, which was mainly focused on birth control through family planning, and formulate a new policy to prevent the problem of aging in the future [18].

### 4.1 Population increase policy requirements

It was found that when policy makers focused on population increase, they considered six general categories of the population policy requirements (Table 2).

**Table 2:** Population growth requirements in Iran according to the previous policies.

Main themes	Sub-themes
Education & acculturation	Public education and attitudinal reform with emphasis on early marriage, the positives of having children, the negatives of low fertility and singlehood.
	Removal of anti-reproductive content from textbooks at all levels of education. Strengthening of physical and mental preparation for responsibility in the family and the desire to have children among students of educational centers.
	Promotion of educations to increase the skills and competences of health professionals to develop natural childbirth**.**
	Strengthening marriage skills and encouraging childbearing in employee groups**.**
	Prohibition of all training against childbearing policies on radio and television, in cyberspace, and in medical centers.
Legal dos and don’ts	Legal prohibition of sterilization and abortion (except for emergency cases) and criminalization and punishment for actions against this law.
	Provision of legal conditions for permanent and temporary remarriage, as well as the abolition of related penalties**.**
	Lowering of the marriage age for women (from 18 years to 13 years) and men (from 20 years to 15 years) and cancellation of sanctions related to marriage with a minor in the Iranian legal system.
	Reducing women’s job and educational opportunities to promote marriage by emphasizing the essential role of motherhood in the family.
	Applying punishment for non-implementation of population increase policies in governmental and non-governmental centers.
Financial and non-financial support for families	Elimination of wage restrictions on children and family allowances and increase of these benefits in proportion to the number of children.
	Allocating special privileges, including priority in employment and improved job security for married couples and people with children.
	Consider flexible work schedules and job privileges for parents with small children.
	Facilitating marriage, housing and childbearing loans and adjusting these loans according to the number of children.
	Construction, improvement and furnishing of student and employee housing with priority for people with children and facilitating housing construction conditions according to the number of children.
	Providing free education, health and nutrition packages for each child and special tax discounts and use of public services based on the number of children.
	Provision of fertility treatment insurance coverage, free treatment insurance for the child and mother, and life insurance for the mother including the minimum premium (free for the poor classes).
	Granting monetary gifts from the government to each child.
Structural and information infrastructure	Establishing and equipping matchmaking, infertility treatment, childbirth, and kindergarten centers to increase the fertility rate.
	Creation of information systems for the purpose of a complete record of information on fertility, infertility, abortion, childbirth, and honoring mothers.
	The organization of the financial mechanisms and the provision of sufficient funding to strengthen population policies.
	Equipping educational centers, and adapting the educational system to strengthen population policies.
	Supporting population research and establishing knowledge-based enterprises consistent with population policies.
Health services	Reducing infertility by implementing extensive infertility screening plans and biological preventive interventions (healthy nutrition and environment).
	Promoting natural childbirth and providing the possibility of high quality natural childbirth.
	Focus on improving maternal and neonatal health indicators through health screening and prevention of high-risk marriages.
	Prohibition of offering all contraceptive methods except those prescribed by a doctor in state medical centers.
Stewardship	Role of the National Population Headquarters in the evaluation, validation, and performance of organizations in line with the implementation of population policies
	Role of the National Population Headquarters in canceling, revising, reforming anti-population laws, and approving new plans and laws for population increase.
	Reduction of activities or closure of national or international organizations working in the field of population control in Iran with the final decision of the National Population Headquarters.

PI1. Education and acculturation**:** This theme (comprising five sub-themes) refers to a series of actions by which policymakers seek to change general attitudes in society. In this regard, aimed at institutionalizing the desired lifestyle, public training should be provided with an emphasis on condemning low fertility and the value of childbearing. Natural childbirth should also be promoted through the mass media, advertising, and the health system. Moreover, educational content opposing fertility policies should be excluded from educational center curricula, while content supporting family formation should be included. On the other hand, childbearing policies should focus on government employees and employees of other institutions as an important part of society.

PI2. Legal dos and don’ts: This theme (comprising five sub-themes) focuses on legal prohibitions, exceptions, penalties for non-compliance with population laws and policies. One of the common approaches is that policy makers try to increase the number of marriages by easing the legal conditions for men to marry and lowering the legal age of marriage. Moreover, the emphasis on the sanctity of mother’s role in the family leads to the adoption of laws that restrict women’s employment and educational opportunities. In these policies, laws related to the criminalization of sterilization and abortion are also regularly updated to prevent declining birth rates.PI3. Financial and non-financial support for families: This theme (comprising eight sub-themes) refers to the provision of financial, and non-financial incentives that are effective in marriage and childbearing. Hence, the government supports married couples and people with children by reducing family-related work restrictions and granting special privileges, including priority in employment and improved job security. Providing cash grants and loans to encourage marriage and providing public housing for married people with children also strengthens the incentive to have children. Besides, the expansion of health insurance coverage, the provision of free health services, and the provision of safe, and sufficient food packages promote healthy population policies alongside population growth policies.

PI4. Structural, and information infrastructure: This theme (comprising five sub-themes) concerns the infrastructure to initiate the population policies. The organization of financial mechanisms, and the provision of sufficient funding for implementing the measures is one of the first points to be considered. Besides, the development and equipment of matchmaking centers, and the establishment of information systems in these facilities is one of the most important infrastructures. The adaptation of educational centers to gender aspects and the creation of effective fields for population growth should be on the agenda. Other aspects of infrastructure that should not be overlooked include financial and operational support for demographic research and knowledge-based enterprises in the field of population.PI5. Health services: This theme (comprising four sub-themes) concerns the provision of health services related to childbearing. Thus, health policymakers focus on infertility screening a healthy nutrition and environment to prevent infertility. Moreover, they pay attention to a healthy birth by conducting health screenings and preventing high-risk marriages. By eliminating the free supply of contraceptives, the goal was to increase the fertility rate and, ultimately, by promoting natural childbirth, the goal was to improve the mother’s health for subsequent births.

PI6. Stewardship: This theme (comprising three sub-themes) refers to the fundamental role of the National Population Headquarters in planning, directing, monitoring, and coordinating all measures related to the population.

### 4.2 Population reduction policy requirements

This study showed that when policy makers were looking for the population reduction, they considered four general categories of the population political requirements (Table 3).

**Table 3:** Population reduction requirements in Iran according to the previous policies.

Main themes	Sub-themes
Religious, scientific, and legal infrastructure	Providing the religious basis in society to initiate a population reduction policy by proclaiming the clear opinion of religious leaders accordingly.
	Engaging the country’s scientific community to begin population reduction efforts by conducting national population seminars and emphasizing maternal and neonatal health indicators.
	Creating an appropriate setting with a positive vote of government board and obtaining legal support to start family planning programs.
Changes in the rules	Changing the points mentioned in occupational laws affecting the number of children (five national occupational laws) consistent with population reduction policies
	Facilitating the legal process of abortion and sterilization and decriminalization of them.
Institution building, programming and division of tasks	Establishment of national macro-centers to carry out the role of guardianship (Birth Control Council) and support (Family Planning Association) and micro-centers to provide family planning services.
	Approving national and international plans and agreements aimed at population control and providing more effective family planning services for the Iranian and non-Iranian populations.
	Approving the duties of the national executive bodies for the implementation of birth control policies by the government board.
	Strengthening of national capacities for conducting general censuses, research and analysis of demographic indicators and their optimal integration into population policies.
Information and service provision	Providing effective educational programs in educational centers with a focus on the artistic community, women and the poor.
	Providing contraceptives with a focus on refugees, the poor and disadvantaged regions.

PR1. Religious, scientific, and legal infrastructure: This theme (comprising three sub-themes) shows how policy makers prepare minds to launch new policies. Religious leaders in Iranian society play a vital role in promoting new policies and ensuring their acceptance in society by supporting and participating in their promotion. On the other hand, to align the scientific community with these policies, the indicators can be provided to study the effect of high fertility on maternal, and infant mortality.

PR2. Changes in the rules: This theme (comprising two sub-themes) highlights the need to review present restrictions in employment laws. Moreover, this theme focuses on the facilitation of abortion and sterilization laws and their decriminalization.

PR3. Institution building, programming, and division of tasks: This theme (comprising four sub-themes) concerns the management of population reduction. First, the implementers of this policy at the macro level and the tasks of government agencies should be specified. At the micro level, the family planning services should be provided. Finally, by the adoption of national and international agreements on population control, all citizens and resident foreigners must also be taken into consideration.

PR4. Information and service provision: This theme (comprising two sub-themes) emphasizes on raising awareness and providing services that lead to the population reduction. Raising public awareness of the problems caused by the population growth should be on the agenda, and in particular efforts should be made to educate illiterate/low-skilled sections of society with local education appropriate to the local culture. Furthermore, the emphasis on raising the educational level of women will increase their participation in society. As part of the policy of population reduction and promoting maternal and child health, educational content should also be changed in educational centers. Another approach is to raise the level of awareness of the artistic community, and create grounds for attracting their active participation in the population reduction. At the same time, education should be provided on using the contraceptives, and in this regard, the government should endeavor to make contraceptives available to members of society, especially in poor and disadvantaged areas.

**Fig. 2:**
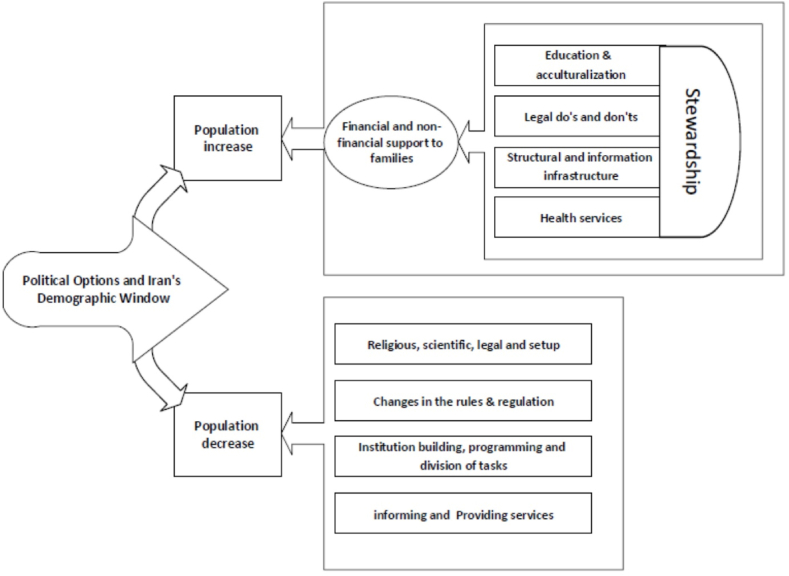
A comprehensive picture of the past population-related policies in Iran.

Finally, Fig. 2 describes the comprehensive picture of the past population policy in Iran. The picture can easily summarize the most important lesson learned from the past. It shows that the family support (financial and non-financial) is the most important element of population increase policies. The effect of such support strongly depends on the presence of related infrastructures. It is evident that the implementation of all policies can only be successful with comprehensive and effective management and good governance. At the same time, the population reduction policies can be successfully formulated, and implemented with a combination of religious, legal, and structural factors and adequate capacity building based on the contextual requirements.

## 5 Discussion

A country with low or negative population growth faces aging populations, slow economic growth, and an unstable healthcare system, while one with a growing population faces undesirable social, economic, and environmental pressures. Each of these countries should adopt policies to prevent pregnancy or deal with low fertility based on their socio-economic condition [51]. The key strategies of policy makers to increase and decrease the Iranian population over 70 years were extensively outlined. Although there are studies on population policies in Iran and the world, no study comparable to this research was found in terms of comprehensiveness in the type of policies reviewed, documents examined, time period, and analysis method. Numerous evidences in the population increase policies indicate that banning contraceptives and abortion is one of the main measures to increase the fertility rate in different countries [52,53]. Scattered studies pointed to the improvement of health infrastructure and the provision of medical treatment, along with financial, and non-financial assistance to families to increase childbearing [[54], [55], [56], [57]]. In general, the evidence showed that policies that reduce the burden of childcare on mothers have a greater impact on childbearing decisions [27]. Furthermore, evidence from Iran shows that to support the policy of population increase, measures such as limiting access to family planning services, increasing maternity leave, and promoting natural childbirth were carried out [2,11,31]. Moreover, the improvement of infrastructure was proposed as the main solution to increase fertility from the point of view of population policy makers in this country [30]. Our comprehensive research has clearly identified that the political requirements to increase the population over the years in Iran have been based on six main themes including “education & acculturation”, “legal dos and don’ts”, “financial and non-financial support for families”, “structural and information infrastructure”, “health services, and stewardship”; and these themes include a total of 30 sub-themes which are discussed in detail in the following sections.

By reviewing several studies in the population reduction policies in Iran and the world, it was found that the formation of relevant institutions to advance the goals of these policies, political-social advertising, informing the community, access to contraceptives, and drugs, removing restrictions on abortion and sterilization, and increasing women’s job opportunities by the purpose of reducing fertility, have been among the most frequent and important measures to achieve the goals of these policies [18,28,[58], [59], [60], [61], [62], [63], [64], [65]]. According to this study, population policy makers in Iran have implemented population reduction policies over the years based on four main themes, including “religious, scientific, and legal infrastructure”, “changes in the rules”, “information and service provision”, and “institution building, programming, and division of tasks”. Below, we explain in detail these themes, which include 11 subthemes. The findings of this research, while covering the results of scattered studies conducted in this field, have exposed the missing links of these studies. Moreover, these results provide policymakers with a detailed description of the previous successful policies with respect to Iran’s socioeconomic and cultural conditions. They can also serve as a model for countries with similar structures and provide a framework for formulating future scenarios. Below, we discussed the themes related to the increase and reduction of population in Iran in two time periods (1978–1988 and 2010–2022), and we have presented evidence of the population policies from other countries.

### 5.1 Population increase: the national slogan “more children, more happiness”

The population increase policies were considered in Iran during two periods. During the first period (1978–1988), almost since the Islamic Revolution, population policies significantly changed in terms of the political developments, changes in value infrastructures, and emergence of religious beliefs [19]. The main strategy of Islamic Republic was to cancel all plans, services, and organizations active in the field of the population control. During this period, the population growth rate increased from 2.6% to 3.9% [66]. During the second period (2010–2022), the country’s tenth president has said that “two children is enough” is a formula for the extinction of a nation, and the country’s officials have expressed concern about the possibility of being overtaken by the aging of population [67]. In this period of time, the “Family Support Plan” and “Population policies announced by the Supreme Leader” were emphasized with the aim of encouraging births and increasing the fertility rate to higher than the replacement level. Moreover, the population growth rate increased from 1.15% to 1.35% [20].

PI1. Education and acculturation (1978–1988): During this period, instead of focusing on the population increase education programs, the government canceled all population control training plans, including house-to-house education, training in educational centers, and training of special groups such as nurses and midwives.

PI1. Education and acculturation (2010–2022): In this period, along with the prohibition of public education against childbearing policies, the Iranian-Islamic lifestyle was emphasized to promote the attitude of community via public education using the mass media and other methods. Educational contents included the negative aspects of divorce, singlehood and having few children, as well as the values of early marriage and having many children. Additionally, all educational levels included trainings aimed at strengthening physical and mental preparation for responsibility and childbearing.PI2. Legal dos and don’ts (1978–1988): After the Islamic Revolution the laws affecting the population increase were changed. Family Planning Act which was lifted and, therefore the minimum age of marriage was severely affected. The legal age of marriage, which before the Islamic Revolution was 18 and 20 respectively for women and men, after the Islamic Revolution, consistent with Islamic rules, was reduced to 13 for women and 15 for men. Moreover, the conditions for permanent and temporary remarriage were eased for men. On the other hand, abortion was allowed only in emergencies, and illegal abortion was followed by severe punishments (up to three years of imprisonment). Furthermore, the employment law led to gender-based job restrictions for women. However, the Iranian constitution did not guarantee the right to education for women despite its emphasis on women’s economic and social well-being. The legal prohibition for women to study in certain fields, and the exclusion of single women from scholarships abroad highlight the educational limitations caused by gender and celibacy in traditional society during this period.

PI2. Legal dos and don’ts (2010–2022): During this time, the law was passed banning sterilization and abortion based on the country’s cultural engineering plan. Permanent sterilization of men, and women or interventions with poor reversibility were prohibited. Sterilization of women was strictly excluded from this prohibition in cases where the pregnancy would endanger the life of the mother. Furthermore, the abortion law was passed much stricter than before. In previous laws, abortion decisions were taken based on the advice of three doctors, but under the new law, abortion decisions are made by a special judge only under certain conditions (abortion is forbidden in cases of doubt).PI3. Financial, and non-financial support for families (2010–2022): All restrictions relating to children and family allowances in the salary of the employee were excluded.

PI3. Financial and non-financial support for families (2010–2022): During this period, the government has provided various support plans for childbearing in the areas of employment, education, housing, insurance, taxes, health, and nutrition. A number of family-related restrictions regarding employment have been removed, and salaries have been increased based on the number of children. Those who were married, and had children had priority for employment and had greater job security. Holidays and education years were increased with each birth and more flexibility was provided in the working hours of parents with young children. On the other hand, private loans for marriage, housing and childbirth were offered and increased based on the number of children. Government housing was provided to students and employees, with priority being given to those with children. Free medical insurance for the child and mother (up to two years), maternal life insurance with minimum premium (free for the poor), and full insurance coverage for infertility treatment were provided. For each birth, the family is offered free educational, health and nutritional packages, and special reductions are granted on taxes and public services (tickets, cinema, and ancient places) depending on the number of children. The government gave monetary gifts to the family for the birth of each child.

PI4. Structural and information infrastructure (1978–1988): Because of the initial crises of the Islamic Revolution and the need for post-war reconstruction, population growth policies attracted great interest during this period. Therefore, all organizations, and sources of funding for population control were suppressed [68]. In this period of time, Iran’s Family Plan was stopped, family welfare centers (which were exclusively focused on the population control plan), and all clinics affiliated with them were handed over to Welfare Organization, mobile units providing family planning services were closed, and Deputy of Health and Family Plan in the Health Ministry was disorganized.

PI4. Structural and information infrastructure (2010–2022): During this period, particular priority was given to the development of infertility services, and the performance of government centers in the implementation of these policies was considered as one of the indicators for funding. In line with Iranian-Islamic culture, marriage facilitation institutions, and free counseling centers were established as part of the marriage promotion approach. The government also established one or more specialist infertility treatment centers in each province and many childbirth centers where women were able to have natural births for free. Besides, to support orphans, 24-h day care centers were set up 1.5 times as before, and qualified child-rearing instructors were hired. The structure of education and research centers was adapted. New fields of study were provided based on the role of the mother in the family in accordance with the Islamic culture, and fields of study related to family planning were removed. The capacity of specialized fields of infertility, obstetrics and gynecology, and other related fields increased. Moreover, an information systems were launched to register information on fertility, pregnancy, abortion, and childbirth. The comprehensive system of “Remote health services for pregnant women” provides free educational and consultation services in health and nutrition during pregnancy and breastfeeding. PI5. Health services (1978–1988): As noted, during this period the government focused more on shutting down family planning services than on providing childbirth services. All contraceptives that were available free of charge prior to this period have been removed.

PI5. Health services (2010–2022): During this period, the government focused on providing childbirth services, banning the free or subsidized distribution of contraceptives in government centers, and banning the sale of non-prescription contraceptives in pharmacies. In addition, special measures were taken to combat infertility. In this regard, food products and environmental pollutants were tested for potential infertility disorders; and a health screening plan was continuously implemented in educational centers. On the other hand, the couples who did not have children were offered screening services for the diagnosis of the cause of infertility and related treatment services.

To improve the health indicators of newborns, a premarital screening certificate (free for the poor) was made mandatory for marriage registration. However, screening for fetal abnormalities could only be done at the request of parents, and with the diagnosis of a specialist. Finally, by providing free natural childbirth in government centers, the focus has been on improving maternal health indicators.

PI6. Stewardship (1978–1988): During this period, the Department of Health and Family Planning, as the main responsible of population control, was completely disorganized which was not replaced by any specific institution.

PI6. Stewardship (2010–2022): The main responsible of population policy during this period was the National Population Headquarters. This headquarters evaluated the performance of organizations related to population policy. It played a central role in the revision of anti-population laws and in the approval of new plans and laws.

### 5.2 Experience of population increase from other countries

A) Chile: After the 1973 military coup, policies called “Re-foundation of the Nation” began and the family planning was canceled. During this period, in terms of these policies and a culture that support male supremacy, women’s control over their own fertility was forbidden and the use of contraceptives was banned by the government. Moreover, illegal abortion was followed by imprisonment [52].
B) Hungary: Since the late 1940s, Hungary has initiated policies to increase fertility and has undergone frequent changes in terms of the power of different political groups. Since 2010, however, it has consistently focused on measures to encourage childbearing [61]. In the 1950s, legal restrictions on abortion and contraception were implemented, although the restrictions were quickly reduced due to public protests [53]. The number of maternity beds and childcare institutions increased [55]. In the 1960s, the amount of child allowance in wage increased in proportion to the number of children and the age limit for abortion was raised to 40 [54]. In the 1980s, wage-adjusted parental leave and tax relief for large families were introduced [56,57]. In 2020, the government offered free infertility treatments in public clinics [57].

### 5.3 Population reduction: the national slogan “Fewer children, better life” and “two children are enough”

Population reduction policies were implemented during two periods in Iran. The first period (1952–1978), before the Islamic Revolution with the national slogan “Fewer children, better life”, which was the first time in the history of Iran, imperial regime started population reduction policies, and the population growth rate was largely controlled [69]. Since the second decade after the Islamic Revolution, the second period (1988–2010) of population reduction began with the national slogan “Two children are enough” and the revival of “Fewer children, better life” [70], and the population growth rate of 4.2% reduced to 1.6% [20].

PR1. Religious, scientific, and legal infrastructure (1952–1978): During this period, the biggest uncertainty was that in Iran, a Muslim country with a culture that believes that children are gifts from God, would family planning work? And is it be accepted by the public? [71]. In reality, policies to reduce the number of children were less approved by the religious authorities. Therefore, the imperial regime first of all tried to gain the support of high-level clerics for these policies.

PR1. Religious, scientific, and legal infrastructure (1988–2010): During this period, due to the consequences of high population growth caused by previous policies, and limited resources, the context was provided to support the population control plan [72]. The conditional support of political and religious leaders legitimized these policies. Consequently, the Supreme Judicial Council issued a statement noting the lack of a legal, and religious ban on family planning.

PR2. Changes in the rules (1952–1978): During this period, the first legal step towards voluntary sterilization in Iran was taken. Consequently, if a necessary surgery is performed according to scientific principles, it is not considered illegal. On the other hand, until then, in the Islamic Penal System, any woman who intentionally caused an abortion was punished with disciplinary imprisonment from one to three years. However, during this period, the mother who performed the abortion was granted parole, and the legal condition was that the mother’s life was in danger.

PR2. Changes in the rules (1988–2010): During this period, the Islamic Penal System was established in relation to sterilization and abortion. In the new law, sterilization was not subject to punishment if it was ineffective. On the other hand, at several stages, the abortion law was established. At first, abortion was only allowed to save the life of the mother and before the soul entered the fetus. Then, the abortion of a fetus with thalassemia major was allowed before the soul entering the fetus. Finally, the country’s abortion commission has broadened the scope of abortion by issuing an ordinance with 49 definitive indications. A change in occupational rules was also made, allowing the salary to increase with an increase in the number of children, up to a maximum of two or three. Besides, insurance, maternity and pregnancy leave and payments related to the cost of raising children for the fourth and subsequent children were abolished.

PR3. Institution building, programming, and division of tasks (1952–1978): During this period, the “Iranian Health and Family Planning Association” was established as the most effective institution for the implementation of population policies and immediately became a member of International Federation of Family Planning Associations. Moreover, the Ministry of Health established the “Supreme Health and Family Planning Council”, “Supreme Coordination Headquarters”, and a new deputy entitled “Population and Family Planning Deputy” according to the suggestion of the experts of the American Population Council. Many small centers have been created to provide services together with national institutions.

PR3. Institution building, programming, and division of tasks (1988–2010): Since the second decade after the Islamic Revolution, population growth reduction has been a priority in the national development plan. In this regard, the “Birth Control Council” and the “General Administration of Population and Family Planning” were established as separate units in the Ministry of Health, and small centers were established to provide family planning services. The macro plan of “Iran Population Control and Family Planning” was approved and for this purpose the legal work of the national institutions was divided. As well as national plans, several international organizations have implemented their own plans, including the World Health Organization, the United Nations High Commissioner for Refugees, the United Nations Population Fund, the World Bank, and UNICEF. PR4. Information and services provision (1952–1978): During this period, the “Health, and Family Planning Association” trained young and literate rural women as “Rural cheerleader”, while providing free contraceptives for the family planning in two general and house to house ways in deprived regions. On the one hand, “welfare centers” which included kindergartens for working mothers and technical-vocational classes for young girls, simultaneously provided family planning services. Besides, in these centers, there were plans to educate men, and sometimes with brief incentives for physicians and young men, group sterilization was carried out. When oral contraceptive methods were introduced to the western world, World Family Planning Federation also sent them to Iran.

PR4. Information and services provision (1988–2010): During this period, public education was provided with the wide role of the artistic society, and the mass media. Training in the educational centers was also focused on groups of reproductive age (especially girls). Through indigenous “health links” who spoke the same language as the poor, education was provided in remote areas to raise awareness. In addition, the government has pledged to provide and distribute free contraceptives via the country. In this regard, the United Nations Population Fund has provided significant assistance. In addition, special attention was given to the poor and family planning services at the household level were provided.

### 5.4 Experience of population reduction from other countries

A) Kenya: Kenya was the first country in sub-Saharan Africa to introduce a population reduction policy [59]. It had almost the highest fertility rate in the world [58]. In 1962, the Family Planning Association of Kenya was established and became a member of International Family Planned Parenthood Federation. In 1965, the government of Kenya invited the “United States-based Population Council” to help the country achieve the optimal rate of population growth [58]. The National Family Planning Council was established in 1967. It has provided volunteer families with information, and supplies for free within the framework of religious prescriptions, through public and private facilities [60]. Information campaigns have improved access to contraceptives through social marketing and community advertising [59].
B) China: Population control policies were gradually initiated in China when total fertility rate reached 6.14 in 1960. In 1953, the State Council passed the “Regulation of Contraception and Induced Abortion” [65]. In 1962, launched an appeal to support family planning and the use of contraceptives [73]. In the 1970s, the supply of contraceptives, and abortion services and the promotion of small families and long intervals between births were expanded, especially in rural areas [65]. Then, one-child policy was introduced which caused a sharp drop in the birth rate [62]. In the 1980s, legal restrictions on family size, late marriages and pregnancy and distance between children were introduced [65]. Forced sterilizations began in the 1990s [74]. Over the next decade, birth control services were provided free of charge [65]. Since 2013, China’s population policy has changed, the national two-child policy was implemented in 2015, and all restrictions were lifted in 2020 [75].

### 5.5 Strengths and limitations

The main strength of this research is its comprehensiveness. Based on the searches, there was no study that analyzed the various published documents (national development plans, related laws, national population management plans, recommendations of Islamic leaders, international support programs, and any other related documents) in 70 years to paint a broad picture of Iran’s population policies. In this research, population policies before the revolution (Pahlavi dynasty) and after the revolution (Islamic Republic), as well as policies aimed at increasing and decreasing the population have been investigated. The main limitation of this study was related to the old documents, in some cases, the original documents were not available and, consequently, the analysis was carried out by referring to secondary references (official websites of the country). Furthermore, in qualitative researches, interviews with experts can provide valuable information regarding the subject under investigation, which was not done in this study. Further research is suggested for determining the consequences of population policies over time to determine their effectiveness, which was not possible in this research. Also, to obtain deeper and more information in this field, it is recommended to conduct more research using a quantitative-qualitative approach based on official census statistics in different time periods and interviewing experts.

## 6 Conclusion

This study, which provides a comprehensive overview of Iranian policies over the past 70 years, has established how population policies have emerged from political-cultural context of society. It shows how these policies laid the foundations for changes demographic structure. The findings showed that the political requirements for population reduction can be classified into four main themes of “Religious, scientific, and legal infrastructure”, “the changes in the rules”, “Institution building, programming and division of tasks”, and “Information and service provision”. Furthermore, the political requirements of increasing population can be divided into six main themes of “Education & acculturation”, “Legal dos and don’ts”, “Financial and non-financial support for families”, “Structural, and information infrastructure”, “Health services”, and “Stewardship”.

## Author contribution statement

Reyhane Izadi, Mohammad Amin Bahrami: Conceived and designed the experiments; Performed the experiments; Analyzed and interpreted the data; Contributed reagents, materials, analysis tools or data; Wrote the paper.

Yaser Sarikhani: Analyzed and interpreted the data; Contributed reagents, materials, analysis tools or data; Wrote the paper.

Peivand Bastani: Conceived and designed the experiments; Analyzed and interpreted the data; Contributed reagents, materials, analysis tools or data; Wrote the paper.

## Data availability statement

No data was used for the research described in the article.

## Funding statement

This research did not receive any specific grant from funding agencies in the public, commercial, or not-for-profit sectors.

## Declaration of competing interest

The authors declare that they have no known competing financial interests or personal relationships that could have appeared to influence the work reported in this paper
