# Supplementary material for: Qualitative document analysis on Iranian contents and trends of population policies: Lessons learned and avenues for future
Source: Heliyon. 2023 Jun 21;9(6):e17377. doi: 10.1016/j.heliyon.2023.e17377 (PMC10319230; doi:10.1016/j.heliyon.2023.e17377)
Supplement: Multimedia component 1 [file mmc1.docx]

| Supplemental Table 1. Name of the documents reviewed in the study | | | |
| --- | --- | --- | --- |
| **No** | **Final Categories of Included Documents** | **Number of Documents** | **Name of documents, year of approval (Solar Hijri year/Gregorian year)** |
| 1 | Social economic development plans (After and before the Islamic Revolution) | 9 | Development plans before the Islamic revolution (Pahlavi dynasty: 1925-1979)   1. Summary collection from the first to the third plan of development 1327/1948-1341/1962 2. The fourth development plan,1347/1968 3. The fifth development plan,1352/1973   Development plans after the Islamic revolution (1979 until now):   1. The first five-year development plan, 1368/1989 2. The second five-year development plan, 1374/1995 3. The third five-year development plan, 1379/2000 4. The fourth five-year development plan, 1384/2005 5. The fifth five-year development plan, 1390/2011 6. The sixth five-year development plan, 1396/2017 |
| 2 | National macro-plans for population management | 9 | 1. Family support plan, 1353/1974 2. Family planning and population plan, 1372/1993 3. The first program of the Family Planning Association of the Islamic Republic of Iran (FPAIRI (, 1379/2000 4. The second program of the Family Planning Association of the Islamic Republic of Iran (FPAIRI (, 1383/2004 5. Women Health Volunteers (as liaisons between the health system and the community) program, 1370/1991 6. Population and workforce plan,1374/1995 7. Infertility treatment service improvement plan,1399/2020 8. The plan to support the family and the youth of the population (Family Excellence Plan), 1400/2021 9. The approval of “National strategies and measures related to preventing the reduction of the fertility rate and improving it in accordance with Islamic teachings and the strategic requirements of the country”, 1391/2012 |
| 3 | Laws and regulations affecting the population management | 11 | 1. Civil Service Management Law 1368/1989, and its amendment 1398/2019 2. The law on the coordinated payment system of government employees 1370/1991, and its amendments in 1375/1996 and 1380/2001 3. The law regulating part of the financial regulations of the government 1370/1991, and its amendments 1380/2001 and 1400/2021 4. Social Security Act 1354/1975, and its amendment in 1400/2021 5. Amendments to the abortion law in 1351/1972, 1384/2005 and 1400/2021 and amendments to the sterilization law in 1350/1976, 1355/1976 and 1400/2021 6. General Penal Code 1352/1973 and Islamic Penal Code 1361/1982, 1370 and 1400/2021 7. The Constitution of the Islamic Republic of Iran, 1358/1979 8. Civil Law of the Islamic Republic of Iran1307/1928, and its amendment 1370/1991 9. Law of the Army of the Islamic Republic of Iran,1366/1987 10. The Disciplinary Force Law of the Islamic Republic of Iran, 1369/1990 11. Guard Corps Law of the Islamic Republic of Iran, 1370/1991 |
| 4 | Serious political recommendations of the leaders of the Islamic Republic of Iran | 2 | 1. The general policies of the population announced by the Supreme Leader (Mod Zaleh Al-Aali), 1393/2014 2. Statements of the founder of the Islamic Republic of Iran regarding population growth in 1358/1979 and 1368/1989 |
| 5 | National land management document | 1 | 1. National and management document,1399/2020 |
| 6 | Duties of communication of executive bodies by the cabinet | 1 | 1. Duties of communication of executive bodies by the cabinet,1386/2007 |
| 7 | International contracts, agreements, and programs in population management | 7 | 1. The financial support program of the World Bank, 1372/1993 2. The World Health Organization's two-year financial support program for maternal-child health and family planning, 1371/1992 3. The first support program of the United Nations Population Fund for the implementation of family planning and population control in Iran, 1369/1990 4. The second support program of the United Nations Population Fund for the implementation of family planning and population control in Iran, 1373/1994 5. The third support program of United Nations Population Fund for reproductive health and family planning in Iran, 1379/2000 6. Cooperation agreement between the United Nations Children's Fund (UNICEF) and the Ministry of Health of Iran,1372/1993 7. The support plan of the United Nations High Commissioner for Refugees (UNHCR) for family planning and reproductive health in two steps inside and outside the camps, 1372/1993 |
